# Application of MXenes in Perovskite Solar Cells: A Short Review

**DOI:** 10.3390/nano11082151

**Published:** 2021-08-23

**Authors:** Syed Afaq Ali Shah, Muhammad Hassan Sayyad, Karim Khan, Jinghua Sun, Zhongyi Guo

**Affiliations:** 1 School of Electrical Engineering & Intelligentization, Dongguan University of Technology, Dongguan 523808, China; ali.shah82@hotmail.com (S.A.A.S.); karim_khan_niazi@yahoo.com (K.K.); sunjh@dgut.edu.cn (J.S.); 2 Advanced Photovoltaic Research Labs (APRL), Faculty of Engineering Sciences, Ghulam Ishaq Khan Institute of Engineering Sciences and Technology, Topi, District Swabi, Khyber Pakhtunkhwa 23640, Pakistan; sayyad@giki.edu.pk; 3 School of Computer and Information, Hefei University of Technology, Hefei 230009, China

**Keywords:** MXene, perovskite solar cells, additives, electrodes, power conversion efficiency

## Abstract

Application of MXene materials in perovskite solar cells (PSCs) has attracted considerable attention owing to their supreme electrical conductivity, excellent carrier mobility, adjustable surface functional groups, excellent transparency and superior mechanical properties. This article reviews the progress made so far in using Ti_3_C_2_T*_x_* MXene materials in the building blocks of perovskite solar cells such as electrodes, hole transport layer (HTL), electron transport layer (ETL) and perovskite photoactive layer. Moreover, we provide an outlook on the exciting opportunities this recently developed field offers, and the challenges faced in effectively incorporating MXene materials in the building blocks of PSCs for better operational stability and enhanced performance.

## 1. Introduction

Sunlight is identified as the most abundant, low-cost and clean energy source for sustainably satisfying the energy requirements of society. Converting sunlight directly to electricity using solar cells is the most efficient and practical way to harness energy from sunlight. Among the various generations of solar cells being developed, those based on Earth-abundant silicon (Si) are dominating the market with power conversion efficiencies (PCEs) of over 26% and an average panel life expectancy of 25 years [1,2,3]. However, the initial fabrication cost associated with Si solar cells is considerably high, shifting the research focus on more affordable alternatives such as dye-sensitized solar cells (DSSCs), quantum dot solar cells (QDSCs), organic solar cells (OSCs) and perovskite solar cells (PSCs).

Among all these emerging PV technologies, PSCs are the most viable alternate offering a comparable PCE to the mature silicon solar cells. In addition, their lower cost, adjustable bandgap, low-temperature solution processability, lower exciton binding energy, high light absorption coefficients, long charge carrier diffusion lengths, multiple options of performance enhancement and significantly simpler mass production techniques bring in added advantages that conventional Si based technologies lack [4,5,6,7,8,9,10]. Furthermore, unlike conventional Si solar cells, PSCs also have high performance in diffused or dim lights, making them ideal for indoor applications [11]. Since the first report on all solid state PSC in 2012, the efficiencies have skyrocketed from 9.7% in 2012 to 25.5% in 2021, much thanks to the development of various architectures, fabrication protocols, advancements in materials, chemical compositions and phase stabilization techniques [12,13,14,15].

Although tremendous progress has been made in achieving state-of-the-art perovskite solar cell, considerable gap still exists between the highest reported efficiency and its theoretical maximum [16,17]. Moreover, questions are raised over stability issues of PSCs, as a thin film solar product must pass the IEC 61646 testing standards, regarding the environmental stability before being commercially viable [18]. Currently, a great deal of research is focused on optimizing the performance and enhancing the stability of PSCs. Various approaches have been reported for performance and stability improvement of PSCs such as use of solar concentrators, spectral modification, bandgap engineering, interfacial passivation and modification, solvent manipulation, additive manufacturing, compositional engineering and use of novel materials [19,20,21,22,23,24,25,26,27,28].

In the last couple of years many groups have reported promising applications of Ti_3_C_2_T*_x_* MXene materials in perovskite solar cells. First discovered in 2011 by Gogotsi and coworkers, transition metal carbides or nitrides (MXenes) emerged as star materials and have shown promising applications in various fields such as energy storage [29,30,31,32,33,34], catalysis [35,36,37,38,39,40], health care [41,42,43,44,45], defense [46,47,48], aerospace [49,50,51,52] and electronics [53,54,55,56]. However, for solar cell applications this field is relatively new as the very first study of MXene materials in perovskite solar cell was reported in 2018, where Guo and group incorporated Ti_3_C_2_T*_x_* as an additive in the photoactive layer of methyl ammonium lead iodide (MAPbI_3_) based perovskite solar cells (PSCs) [57]. Since then, MXene fever has taken the photovoltaic community by storm and its applications have extended from simple additives in the photoactive layer and HTL/ETL to being used as electrode and HTL/ETL.

**Figure 1:**
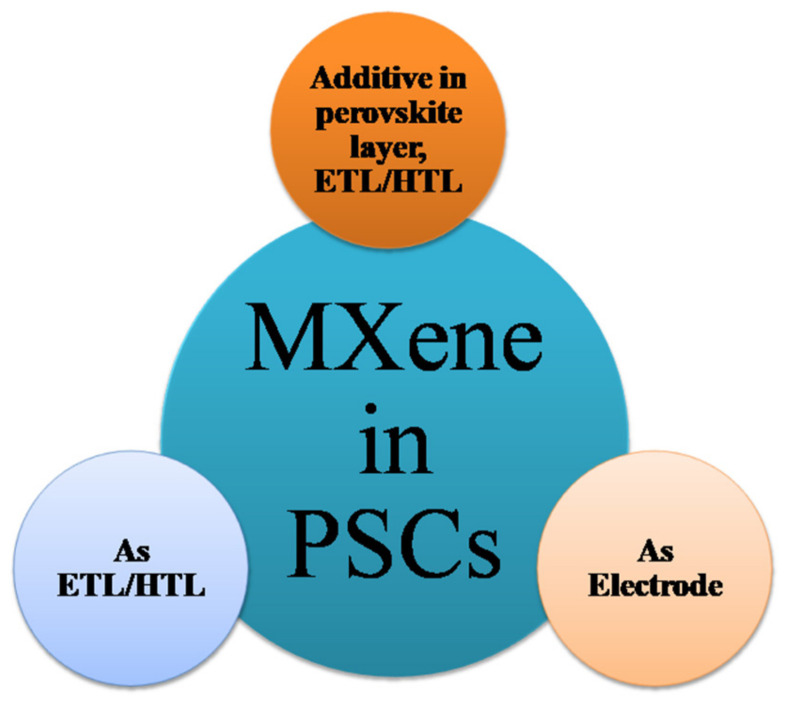
Incorporation of MXene in PSCs.

In view of the tremendous research interest attracted by use of Ti_3_C_2_T*_x_* MXene in perovskite solar cells; this short review aims to summarize all the work reported so far on the incorporation of MXene in PSCs to improve solar energy harvesting and enhance operational stability. We begin with a quick overview of the most common architectures of PSCs. Moving forward, the focus of this review then shifts towards understanding all the possible interactions of 2D MXenes within the PSCs by highlighting all the experimental and theoretical studies reporting the incorporation of MXene in PSCs, with special emphasis being placed on classifying them according to the major role these materials play in PSCs such as additives, electrodes and HTL/ETL as shown in Figure 1. Furthermore, an outlook on the opportunities this emerging field offers, and the challenges faced in effectively using MXene materials in PSCs for improved operational stability and enhanced performance is provided.

## 2. Structure of PSCs

**Figure 2:**
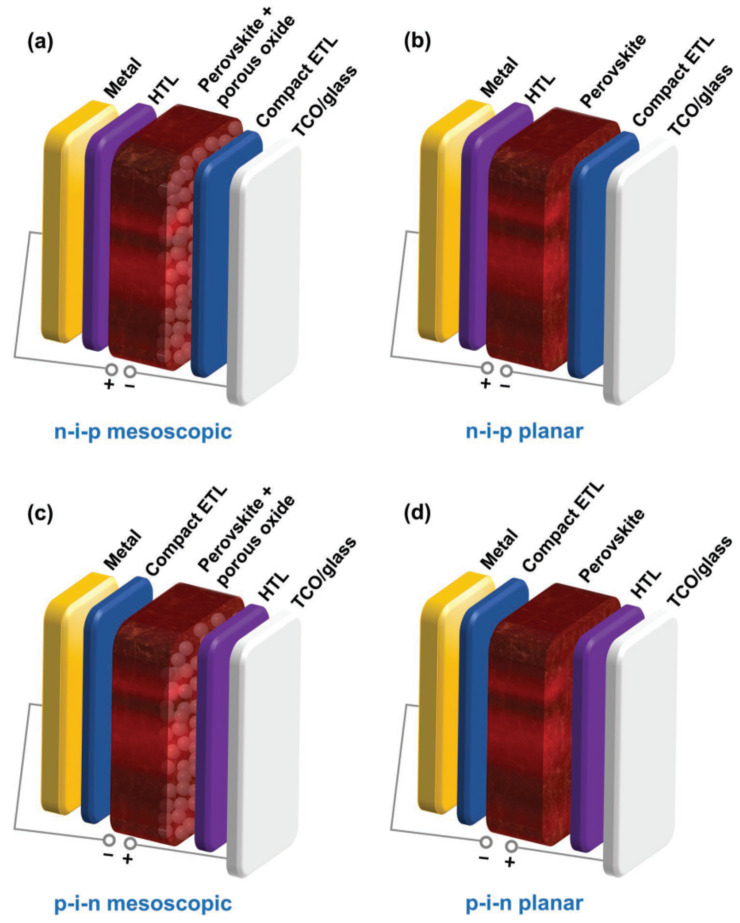
Four typical PSC architectures. Reprinted with permission from Ref. [58]. Copyright 2019 WILEY-VCH Verlag GmbH & Co. KGaA, Weinheim.

PSCs can broadly be divided into two categories, the regular (n-i-p) structure and the inverted (p-i-n) structure as shown in Figure 2. This classification of PSCs is based on the charge transport layer (ETL/HTL) that is first encountered by the incident light. These two types of architectures can further be divided into mesoscopic and planar structures, respectively. The mesoscopic structure incorporates additional mesoscopic layer acting as a scaffold for the perovskite absorber to improve the charge collection, whereas the planar structure consists of all planar layers [58]. Mesoscopic devices have structural similarity with typical dye sensitized solar cells (DSSCs) and are the dominating device architecture of PSCs. However, unlike DSSC a much thinner mesoporous layer is used in PSCs, where the photoactive perovskite layer fills the interstice of mesoporous charge-transporting layer before forming an overlayer on top of it. Compared to the complex mesoscopic architecture, the planar structure of PSCs is much simpler and is better suited for flexible devices because of its low temperature processing. Planar structure uses multiple stacked thin layers, such as the photoactive perovskite layer is sandwiched between the HTL and ETL. As the detail investigations of basic principles, fabrication protocols, materials and methods of PSCs are beyond the scope of current study, for more details we encourage readers to refer to recently published reviews [59,60,61].

## 3. Applications of MXenes in PSCs

### 3.1. MXene as Additive in Photoactive Perovskite Layer, ETL and HTL of PSCs

Despite rapid improvements in the PCE, the performance of PSC is still limited by excessive charge carrier recombinations inside the photoactive perovskite layer and at perovskite/ETL, perovskite/HTL interfaces. To bridge the gap between current PCE and the theoretical efficiency limit of PCSs, improvements in charge carrier management is critical. In addition, the inherent instability of perovskite in moisture and elevated temperatures and poor device scalability must be overcome before mass production. In recent years 2D nanomaterials with unique properties have been explored as additives in photoactive perovskite layer or ETL/HTL of PSCs. To improve the crystallization and surface coverage of perovskite films, additive engineering has proven to be an effective method. For example, Hagfeldt and coworkers incorporated nitrogen doped graphene into the photoactive layer of PSCs for affectively increasing the perovskite grain size and reducing recombinations through surface passivation [62]. Other such fascinating recently explored 2D materials for PSCs applications include g-C_3_N_4_ [63], WS_2_ [64], MoS_2_ [65] and black phosphorus [66].

**Figure 3:**
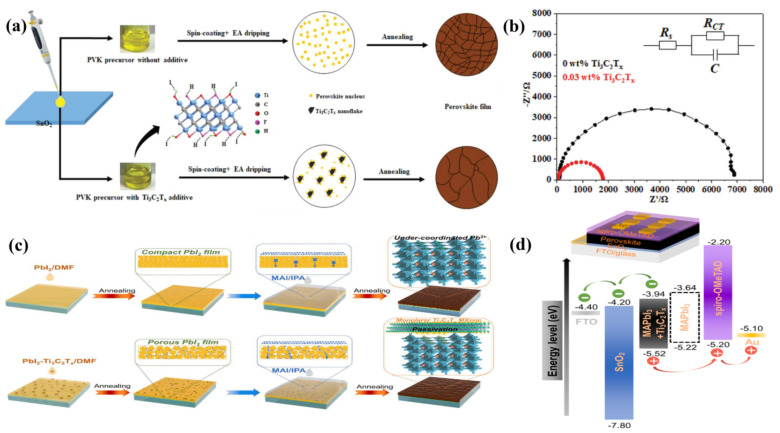
(**a**) Proposed nucleation and growth route of perovskite film with and without Ti_3_C_2_T*_x_* additive (**b**) Nyquist plots of 0 and 0.03 wt% Ti_3_C_2_T*_x_* additive–based device measured in the dark with a bias of 0.7 V. Reprinted with permission from Ref. [57]. Copyright 2018 WILEY-VCH Verlag GmbH & Co. KGaA, Weinheim. (**c**) Proposed mechanism of preparing high-quality two-step-processed Ti_3_C_2_T*_x_* added perovskite films (**d**) Device architecture and energy level alignment. Reprinted with permission from Ref. [67]. Copyright 2020 Elsevier B.V. All rights reserved.

Guo et al., were the first to report Ti_3_C_2_T*_x_* as an additive into the photoactive layer of PSCs (Figure 3a) [57]. The termination groups of Ti_3_C_2_T*_x_* slowed the perovskite crystallization rate, resulting in increased crystal sizes. Moreover, the superior electrical properties of Ti_3_C_2_T*_x_* enhanced the charge transfer dynamics of PSCs as can be seen in Figure 3b, to achieve a power conversion efficiency (PCE) of 17.41% with J_SC_ of 22.26 mA cm^−2^, which corresponded to 12% and 7.6% increase in PCE and J_SC_, respectively, with respect to the control device (PCE of 15.54% and J_SC_ of 20.67 mA cm^−2^). This increase in PCE and J_SC_ was attributed to the high electrical conductivity of MXene that resulted in superior charge transfer dynamics, faster electron extraction and reduced recombinations. As can be seen from Figure 3b, the charge transfer resistance R_CT_ reduced from 7000 to 1800 Ω signifying the considerable increase in conductivity of perovskite film upon Ti_3_C_2_T*_x_* MXene incorporation.

One of the most important possible applications of 2D materials is the ability to easily modify their electronic structure, such as bandgap or work function (WF) via suitable functionalization. The WF can be fine-tuned as per need resulting in optimal energy level alignment for a perfect energy offset between the perovskite active layer and ETL/HTLs, eventually inducing built-in potential for efficient charge transport dynamics [68]. Agresti et al., incorporated Ti_3_C_2_T*_x_* MXene in MAPbI_3_ PSCs to favorably adjust the WF of perovskite film and ETL, improving the PCE by 26% as compared to Ti_3_C_2_T*_x_* free control devices [68]. Di Vito and group performed first-principles calculations based on DFT on Ti_3_C_2_/MAPbI_3_ perovskite coupled system and linked the WF tuning with varying the relative concentrations of OH, O, and F MXene terminations, and concluded that OH groups have the strongest effect in lowering of WF [69].

Recently, Zhao et al., reported MXene nanosheets as multifunctional additive into PbI_2_ layer during the first step of CH_3_NH_3_PbI_3_ formation. Such an introduction of MXene nanosheets into PbI_2_ facilitated a complete transformation of both reactants (PbI_2_ and CH_3_NH_3_I) into CH_3_NH_3_PbI_3_ during the second step of MAPbI_3_ formation, leaving no residue PbI_2_ in the perovskite film and enlarging the grain size of perovskite [67]. In addition, the incorporation of Ti_3_C_2_T*_x_* nanosheets in perovskite layer increased the WF of MAPbI_3_ by 0.3 eV shifting it from 3.82 eV to 4.12 eV, rendering the conduction band (CB) of MAPbI_3_-Ti_3_C_2_T*_x_* much closer to the CB of the ETL as can be seen from Figure 3d, resulting in efficient electron extraction. As a result of the multiple enhancement effects of the Ti_3_C_2_T*_x_* MXene nanosheets introduction, the optimized device with Ti_3_C_2_T*_x_* dosage of 0.03 wt%, achieved a champion PCE of 19.27% with a V_OC_ of 1.12 V, a J_SC_ of 23.48 mA cm^−2^ and a FF of 0.736, which corresponded to almost 18%, 3.7%, 9% and 4.3% increases in the PCE, V_OC_, J_SC_, and FF, respectively, as compared to the pristine PSCs (16.54%).

**Figure 4:**
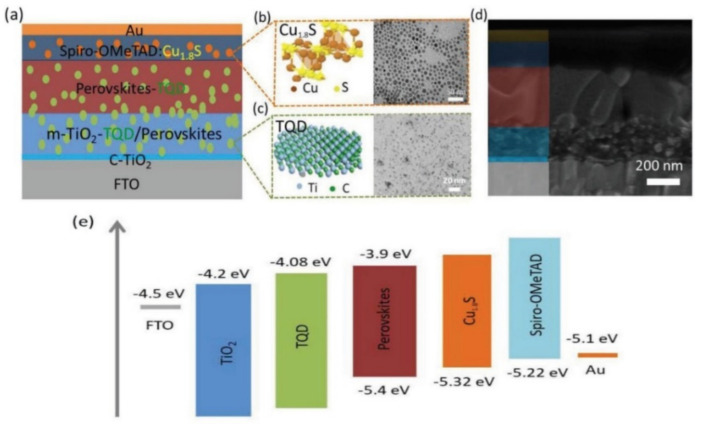
(**a**) PSC architecture. (**b**) Schematic structure and TEM image of Cu_1.8_S. (**c**) Schematic structure and TEM image ofTi_3_C_2_ QDs. (**d**) Cross-sectional SEM of a complete device. (**e**) Energy diagram of each layer in PSCs. Reprinted with permission from Ref. [71]. Copyright 2020 WILEY-VCH Verlag GmbH & Co. KGaA, Weinheim.

In 2020, Zhang and group reported the synthesis of MAPbBr_3_ nanocrystals (NCs) on the surface of few-layer MXene (Ti_3_C_2_T*_x_*) nanosheets forming heterostructures employing an in situ solution growth method [70]. The heterostructures, ensured efficient electron injection from the MAPbBr_3_ NCs to the Ti_3_C_2_T*_x_* MXene because of the well aligned energy levels. In a first for its kind, Chen et al., utilized ultrathin Ti_3_C_2_T*_x_* quantum dots (TQDs) for dual interfacial modifications, simultaneously engineering the photoactive absorber and the perovskite/TiO_2_ ETL interface, as can be seen in Figure 4a [71]. PSCs were fabricated employing c-TiO_2_/m-TiO_2_-TQD/TQD-perovskite/Spiro-OMeTAD-Cu_1.8_S architecture to enhance the PCE and stability of devices simultaneously achieving a champion hysteresis-free PCE of 21.64% with significantly higher long-term air and light stability compared to 18.31% for control devices. The electrochemical impedance spectroscopy (EIS) study of devices revealed that addition of TQD in perovskite layer and ETL significantly increased the charge recombination resistance (R_rec_) from 613 Ω for control device to 993.7 Ω for optimized device. The improved performance was attributed to several factors such as suppressed charge carrier recombination because of the well-matched energy levels (Figure 4e), facilitating electron extraction efficiency at the semiconductor/perovskite interface and reduced trap state densities within the perovskite film, resulted from the effective passivation of TQD toward grain boundaries.

As a new field, the full potential of MXene materials in PSCs is yet to be explored. However, different groups reported unique strategies of benefitting from the superior electrical conductivity offered by 2D MXene materials. One such attempt was made very recently by Zhao and group, reporting a charge behavior modulation in PSCs by simultaneous use of 2D Ti_3_C_2_ nanosheets and 0D Ti_3_C_2_ QDs in ETL and perovskite layer respectively [72]. While the introduction of Ti_3_C_2_ QDs into the perovskite layer effectively passivated the defects of perovskite film, addition of Ti_3_C_2_ nanosheets into the TiO_2_ ETL significantly increased the electron mobility and extraction rate. Hence, the device based on 0D Ti_3_C_2_ QD–modified photoactive layer and 2D Ti_3_C_2_ nanosheets-modified TiO_2_-ETL achieved a PCE of 17.1%, while the control device only delivered a PCE of 12.0%. Jin et al., introduced 2D Ti_3_C_2_T*_x_* MXene nanosheets as nanosized additives in 2D Ruddlesden-Popper PSCs. Thanks to the enhanced crystallinity, optimized orientation, lower charge transfer resistance and passivated trap states, the PCE, V_OC_, J_SC_ and FF of 2D PSCs increased from 13.69%, 1.09 V, 18.84 mA cm^−2^, and 66.7% (control device without any MXene additive) to 15.71%, 1.11 V, 20.87 mA cm^−2^ and 67.84%, respectively [73].

Yang et al., reported SnO_2_-Ti_3_C_2_ MXene nanocomposites as electron transport layers (ETLs) in planar PSCs [74]. SnO_2_ with different Ti_3_C_2_ MXene weight ratios (0, 0.5, 1.0, 2.0, 2.5 wt.%) was used as ETL and PSCs with ITO/ETL/MAPbI_3_/Spiro-OMeTAD/Ag configuration were fabricated. The addition of Ti_3_C_2_T*_x_* enhanced the conductivity of ETL, facilitating electron transport. Moreover, the addition of Ti_3_C_2_T*_x_* optimized the energy level alignment further enhancing charge transport dynamics and reduced recombinations. The optimized device with 1.0 wt.% Ti_3_C_2_ achieved overall PCE of 18.34%, corresponding to an increase of 6.4% compared to the PCE of control device without Ti_3_C_2_T*_x_* addition (17.23%).

**Figure 5:**
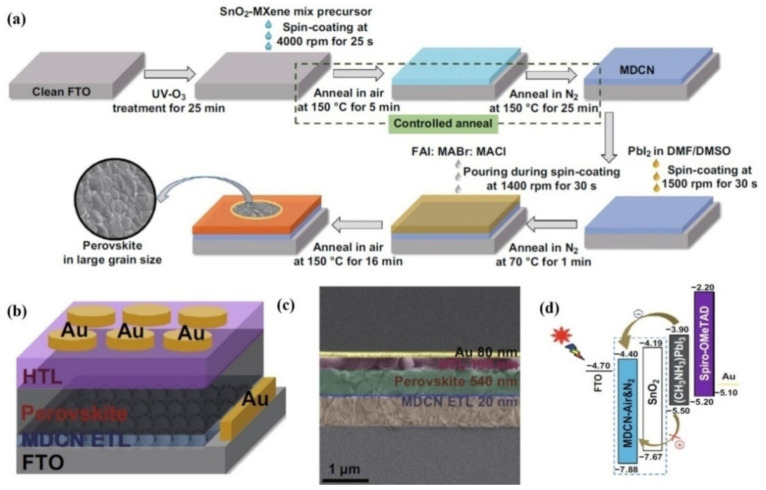
(**a**) Synthesis process for perovskite layer with MDCN ETL (**b**) PSC structure employing MDCN as ETL (**c**) Cross-sectional SEM image of a complete device with MDCN-0.02 ETL (**d**) Energy diagram for a working device with potential ETLs signed in the dotted box Ref. [75].

Huang and coworkers took the work of Yang et al., a step further by surrounding the 2D Ti_3_C_2_T*_x_* sheets with 0D anatase TiO_2_ QDs to form effective TiO_2_/SnO_2_ heterojunctions, which they named a multi-dimensional conductive network (MDCN) structure, as can be seen in Figure 5b,c [75]. Due to the favorable energy-level alignment (Figure 5d) between the ETL, mix cation (FAPbI_3_)_0.97_(MAPbBr_3_)_0.03_ perovskite and the FTO, an enhancement of PCE from 16.83% to 19.14% was achieved. Moreover, the V_OC_, J_SC_ and FF increased from 1.07 V, 24.07 mA cm^−2^ and 73.61 to 1.1 V, 24.52 mA cm^−2^ and 77.97, respectively. In addition, the MDCN-incorporated device exhibited superior moisture-resistance and retained almost 85% of the initial performance even after 45 days in 30–40% humidity. This superior stability was attributed to an oxygen vacancy scramble effect.

Very recently, Yang et al., reported the modulation of perovskite crystallization rate using MXene QDs-modified SnO_2_ ETL [76]. The Ti_3_C_2_T*_x_* QDs-modified SnO_2_ (MQDs-SnO_2_) ETL effectively modulated the crystallization rate by rapidly inducing perovskite nucleation from the precursor solution, producing an intermediate phase upon anti-solvent treatment. This process considerably improved the crystal quality and enhanced the phase stability of the as-fabricated perovskite film. Moreover, the addition of MXene QDs in SnO_2_ added extra advantage of superior charge extraction of the ETL, providing a PCE of up to 23.3%, with outstanding stability of over 500 h against humidity and light soaking. The same group also reported using Ti_3_C_2_T*_x_* nanosheets incorporated TiO_2_ as ETL for Cs_2_AgBiBr_6_ double-PSCs [77]. Not only did the incorporation of Ti_3_C_2_T*_x_* nanosheets in TiO_2_ improved the electrical conductivity and electron extraction of ETL, but also modified the surface wettability of ETL to promote optimized and efficient crystallization of double perovskite layer. Owing to these beneficial effects the modified device yielded a hysteresis free PCE of 2.81% and remained stable for more than 15 days under ambient conditions.

**Figure 6:**
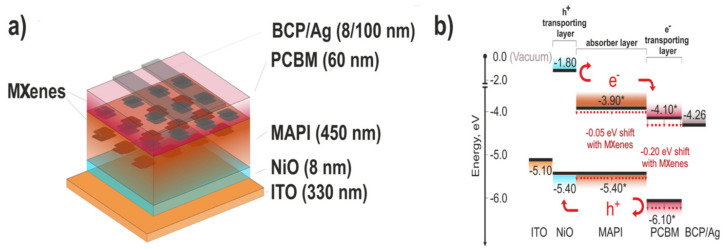
(**a**) Schematic representation of MXene-based p-i-n PSCs (**b**) energy level diagram for a PSC. Reprinted with permission from Ref. [78]. Copyright 2021 Elsevier Ltd. All rights reserved.

In 2021, Saranin and coworkers doped MXenes into both the photoactive perovskite layer and ETL in NiO-based p-i-n PSCs, simultaneously tuning the energy level alignment at perovskite/charge transport layer interfaces and passivating the traps states within the device, resulting in improved charge collection/extraction at the electrodes. PSCs having NiO/MAPbI_3_/PCBM/bathocuproine (BCP)/Ag structure were fabricated with Ti_3_C_2_T*_x_* MXene doped in both PCBM and perovskite layer (Figure 6a). Thanks to the optimized energy level alignment (Figure 6b) and suppressed trap states, the MXene-based improvised devices achieved superior performance, with 19.2% PCE and significantly improved stabilized power output as compared to control devices [78].

Incorporating Ti_3_C_2_T*_x_* into the HTL can also have a profound impact on device performance as was demonstrated recently by Hou et al., for organic solar cells (OSCs). Where addition of Ti_3_C_2_T*_x_* MXene into the HTLs improved device performance by 11% [79]. Not only did the addition of Ti_3_C_2_T*_x_* MXene into PEDOT:PSS achieved better PCE, it also improved the long-term stability compared to Ti_3_C_2_T*_x_* free based devices. Although these results prove the promising prospects of PEDOT:PSS/Ti_3_C_2_T*_x_* composite films in highly efficient and stable photovoltaic devices, to the best of our knowledge, no such study has been undertaken for PSCs to explore the addition of MXene into HTL of PSCs. The use of 2D MXene materials as additives in perovskite active layers and ETL/HTL is still in its early stages of development. There’s still a lot to be done in order to enhance the PSC efficiency. However, we believe a significant breakthrough in this cutting-edge technology can be expected in the near future.

### 3.2. MXene as an ETL/HTL in PSCs

Electron transport layer (ETL) and hole transport layer (HTL) play an important role in improving the photovoltaic performance and stability of perovskite solar cells (PSCs). The ETL essentially has the vital role of collecting and transferring the electrons from the perovskite layer and block the backflow of holes, effectively separating charges and suppressing charge recombination [80]. The major role of HTL is to extract and transport the holes from the photoactive perovskite layer to the electrode, simultaneously acting as an energy barrier preventing the transfer of electrons to the anode. In addition, the HTL effectively separates the photoactive perovskite layer from the anode and isolates the moisture in the air, enhancing the stability of PSCs by suppressing degradation and corrosion [59]. The charge selective transport layers are critical not only in the extraction and transport of charge carriers, but also in the optimization of perovskite thin film growth in those high-efficiency PSC devices. Furthermore, they are critical to the scalability and stability of devices. In general, efficient PSCs have two key interfaces, such as the ETL/perovskite heterojunction interface and the perovskite/HTL interface, which should both ensure effective charge collection and separation [81]. Inefficient charge transport can result in inhomogeneous charge accumulation and significant interfacial recombination, whereas a high-quality ETL and HTL not only collects and transfers charge carriers, but also effectively separates charges and suppresses charge recombination. As a result, it is crucial to develop and manufacture high-quality ETLs and HTLs in order to ensure efficient charge transfer and consequently ensure efficient photovoltaic performance of devices.

Due to the outstanding intrinsic properties of Ti_3_C_2_T*_x_* MXene material, such as very high conductivity, adjustable surface functional groups and easily tunable WF, its potential application as ETL/HTL in PSCs have recently attracted significant research interest. In 2019, Chen et al., reported a novel architecture incorporating the Ti_3_C_2_-MXene into the CsPbBr_3_ perovskite to fabricate a HTL-free inorganic PSC with (FTO/TiO_2_/CsPbBr_3_/Ti_3_C_2_-MXene/C) structure. The 2D Ti_3_C_2_-MXene interlayer served the function of HTL by ensuring improved energy level alignment between the perovskite film and carbon electrode and accelerated the hole extraction. The introduction of Ti_3_C_2_-MXene significantly enhanced the hole extraction while simultaneously blocking the electron from reaching counter electrode. Additionally, the functional groups of Ti_3_C_2_T*_x_* well passivated the CsPbBr_3_ grains, resulting in reduced trap defects in the perovskite film. The cell without Ti_3_C_2_-MXene interlayer achieved a poor PCE of 8.21% with V_OC_ of 1.423 V, a J_SC_ of 7.96 mA cm^−2^ and a FF of 72.48%. Increased charge carrier recombinations at the interface were identified as the primary cause of lower efficiency. In contrast, thanks to a much reduced energy level mismatch, enhanced hole extraction rate and suppressed recombinations, the CsPbBr_3_/Ti_3_C_2_-MXene based device achieved a high PCE of 9.01% (Voc of 1.444 V, Jsc of 8.54 mA cm^−2^ and FF of 73.08%) with a surprising long-term stability of over 1900 h [82].

**Figure 7:**
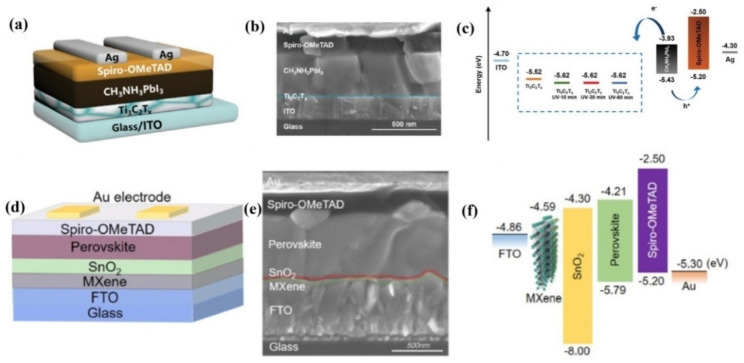
(**a**) Device architecture of based on Ti_3_C_2_T_x_ with UV-ozone treatment as ETL (**b**) cross sectional SEM image of the PSC device (**c**) schematic energy-level diagram of each layer. Reprinted with permission from Ref. [83]. Copyright 2019 WILEY-VCH Verlag GmbH & Co. KGaA, Weinheim (**d**) Device configuration. (**e**) Cross-sectional SEM image of device. (**f**) Flat-band energy level diagram. Reprinted with permission from Ref. [84]. Copyright 2020 American Chemical Society.

In the same year, Yang et al., reported the use of surface-modified metallic Ti_3_C_2_T*_x_* MXene as ETL for planar PSCs, as can be seen from Figure 7a,b [83]. Using UV-ozone treatment, the WF of Ti_3_C_2_T*_x_* MXene was downshifted from −5.52 to −5.62 eV, as shown in Figure 5c. Devices fabricated employing 30 min UV-ozone treated Ti_3_C_2_T*_x_* nanosheets as ETL resulted in a high PCE of 17.17%. It was observed that the UV-ozone treatment of Ti_3_C_2_T*_x_* improved the Ti_3_C_2_T*_x_*/perovskite interface properties because additional oxide-like Ti–O bonds were formed on the surface of Ti_3_C_2_T*_x_*. In addition, the downward shifting of WF because of the treatment, made the Ti_3_C_2_T*_x_* more efficient towards transmitting electrons and blocking holes, eventually reducing recombination, leading to much higher PCE than those without the UV-ozone treatment. EIS was used to gain better insight into the charge transport mechanism of the devices, which revealed the smallest charge transfer resistance of 27.86 Ω for the device with 30 min UV-ozone treated Ti_3_C_2_T*_x_* as compared to all other devices.

Very recently, Yang and group used a similar approach of WF modifications by oxidation of Ti_3_C_2_T*_x_* in air and employing them as ETL in PSCs [85]. Oxidation of Ti_3_C_2_T*_x_* formed Ti–O bonds significantly improving the interface properties of the MXene/perovskite junction and reduced the macroscopic defects in the photoactive layer spin coated ontop of MXene layer. At light oxidation, while the metallic character of Ti_3_C_2_T*_x_* remained similar, the WF shifted from −5.35 to −6.88 eV, owing to the newly formed TiO_2_. However, at heavy oxidation a transition from metallic material to semiconductor was observed. When PSCs were fabricated using a mix of oxidized and pristine Ti_3_C_2_T*_x_* as ETL, a much better alignment of energy levels between ETL and perovskite layer was obtained, rendering a very high PCE of 18.29%.

In 2020, Wang et al., reported a champion stabilized PCE of 20.65% with a V_OC_ of 1.11 V and highly improved J_SC_ of 24.34 mA·cm^−2^, by introducing a thin layer of Ti_3_C_2_T*_x_* MXene between the SnO_2_ ETL and electrode of PSC [84]. As can be seen from Figure 7f, the introduction of Ti_3_C_2_T*_x_* MXene layer between SnO_2_ ETL and electrode optimized the conduction band alignment, resulting in improved charge transfer kinetics and better performance. A lower series resistance of 2.98 Ω.cm^−2^ was obtained for MXene-modified device compared to the 3.84 Ω.cm^−2^ of control device. Furthermore, the incorporation of MXene resulted in much smoother and hydrophobic surface of SnO_2_ ETL compared to SnO_2_ surface without underlying MXene, thus facilitating growth of uniform and defect free perovskite films with significantly low trap density. Thanks to the synergetic effects introduced by MXene layer, such as significantly reducing non-radiative recombinations, improving homogeneity and suppressing carrier transport loss, the fabricated device achieved a stabilized PCE of 20.65%, which was almost 8.6% higher than that of the control device without MXene thin layer (19%).

To suppress MXene oxidation at elevated temperatures and further improve the conductivity of MXene within the PSC, very recently, Bati et al., introduced MXene-CNTs hybrids between SnO_2_ ETL and perovskite layer [86]. It was revealed that the synergetic effects introduced by MXene and metallic SWCNTs hybrids and the reduced trap density resulted from introduction of MXene-CNTs hybrids can significantly optimize the SnO_2_/perovskite charge dynamics. When the ratio of MXene/m-SWCNTs was kept at 2:1 w/w, a champion PCE of 21.42% was recorded with an astonishing increase observed in fill factor (FF) from ≈0.69 to ≈0.80, whereas the R_CT_ decreased substantially from 165 Ω to 33.5 Ω. From the above discussion it is evident that MXene material is an ideal substitute for the ETL and HTL materials currently in use for PSCs. The use of MXene not only proved beneficial for a fast charge collection/extraction dynamics, hence providing a much better PCE, it also effectively isolated the perovskite layer from the humidity in turn improving the stability and shelf life of devices.

### 3.3. MXene as an Electrode in PSCs

An electrode is one of the major constituents of a PSC governing the process of charge collection, playing a key role in long term stability, and determines the total cost of device. Some of the recently reported electrode materials for PSCs include metal thin-film electrode, nano-structured metal electrode [87], graphene electrode [88] and carbon electrodes [89,90]. Ti_3_C_2_T*_x_* MXene emerged as the most suitable choice for potential application as electrode in PSCs because of its many excellent properties such as high intrinsic electrical conductivity [91], high optical transparency [92], outstanding biocompatibility, remarkable flexibility, environment-friendliness and adjustable WF [93].

**Figure 8:**
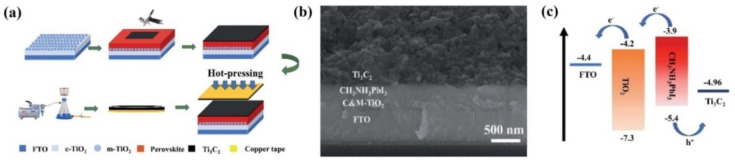
(**a**) Fabrication process of Ti_3_C_2_ electrode-based PSC employing hot-pressing technique. (**b**) Cross-sectional SEM image (**c**) Representative energy-level alignment of the PSC. Reprinted with permission from Ref. [94]. Copyright 2019 The Royal Society of Chemistry.

In 2019, Cao et al., reported the very first use of 2D MXene material (Ti_3_C_2_) as a back electrode in hole transport materials and noble-metal-free PSCs [94]. A simple hot-pressing technique was employed to form a direct interfacial contact between the MAPBI_3_-photoactive perovskite layer and the Ti_3_C_2_ MXene electrode as can be seen in Figure 8a. As is evident from Figure 8c, the WF of Ti_3_C_2_ electrode (4.96 eV), matched very well with the valence band of MAPbI_3_, facilitating charge transfer. After careful optimization of the Ti_3_C_2_ electrode, a high PCE of 13.83% with a Voc of 0.95 V, Jsc of 22.97 mA cm^−2^, and FF of 63% was obtained, which corresponded to almost 27% increase in PCE compared to that of the reference devices using carbon electrodes. This enhancement in PCE was attributed to a much better charge extraction capability and a much lower square resistance of the Ti_3_C_2_ electrode compared to the carbon electrodes. Moreover, the seamless interfacial contact between the MXene electrode and the MAPBI_3_ perovskite film also helped improve the overall stability of PSC based on MXene electrode.

Very recently, Jiang and group reported a mixture of 1-D carbon nanotubes (CNTs), 2D Ti_3_C_2_-MXene nanosheets and commercial carbon paste as electrode material in CsPbBr_3_-PSC to achieve a respectable PCE of 7.09% [95]. Not only did the introduction of CNTs and MXene effectively enhance the conductivity, it also provided a multi-dimensional route for improved charge extraction and transport dynamics. Table 1 summarizes all the work reported till date on the incorporation of 2D-MXenes in the building blocks of perovskite solar cells. From the above discussion and Table 1, it can be concluded that introduction of 2D-MXenes (Ti_3_C_2_T*_x_*) as additive in perovskite layer and ETL/HTL or its use as ETL/HTL and electrode causes improvement of the chemical and electrical properties of the PSCs due to their high conductivity, WF adjustment capability and improved charge collection/transport properties. The Ti_3_C_2_T*_x_* also resulted in uniform and smooth perovskite films with minimum trap state density, leading to improved performance with minimum recombinations. Furthermore, in few cases the Ti_3_C_2_T*_x_* layers helped isolate the sensitive perovskite layer from outside humidity, considerably improving the shelf life and device stability upon prolonged illumination.

## 4. Prospects and Challenges

Application of MXenes in perovskite solar cells (PSCs) is an emerging field and has opened a wide spectrum of research. Despite many significant developments, we believe the reported work only represents the tip of the iceberg and many important discoveries are on their way. Given the fact that within few years, more than 20 different MXenes have been produced, many more can be expected in the coming years, with different compositions, various surface terminations, variable band structures and adjustable work functions. The possibilities are countless, promising huge potential for PSCs applications.

Going forward, we believe, apart from the experimental work reported so far, more theoretical work is needed in order to gain insight into charge transport/extraction behavior and work function modification of new MXene compositions, MXene/perovskite, MXene/ETL and MXene/HTL interfaces. Detailed investigations are desired on the impact of MXene surface modifications, various termination groups, work functions modifications and energy level alignment on the photovoltaic parameters of MXene-based PSCs.

MXene dopant concentration has a profound impact on the photovoltaic performances of PSCs; therefore, the doping concentration must be optimized. For very low concentrations no improvement is achieved in the uniformity and defects passivation of perovskite films. However, a too high dopant concentration can adversely affect the quality of perovskite films and result in a rougher film. Moreover, an optimized concentration is necessary to achieve good crystallization of perovskite films such that the MXene must be homogeneously distributed along the longitudinal and transverse directions of the perovskite film.

The electronic structure of MXenes is dictated by the functional groups on the surface, inevitably shifting the WF. An optimized energy-level alignment between the perovskite and ETL can ensure effective charge transfer dynamics while simultaneously suppressing the recombinations in the inner perovskite layer and at the different interfaces involved in PSCs. A thorough investigation into understanding the fascinating phenomenon of work function modification is required to unravel the effects of parameters such as synthesis conditions, post-synthesis thermal/UV-Ozone treatment and chemical treatment.

Apart from the good flexibility and high electrical conductivity, MXenes should be transparent in the photovoltaic response range of PSCs. Moreover, for commercial applications of MXene in PSCs various issues must be considered, such as environment friendly incorporation techniques that allow large scale production and stability under ambient conditions. Beside the advantages offered by MXene in terms of material qualities and performance, methods and procedures connected to fabrication of MXene must ensure competitive pricing, environmental friendliness, long-term reliability and sustainable manufacturing.

## 5. Conclusions

The last decade has witnessed dramatic technological advancements in perovskite solar cells (PSCs). The transition from traditional architectures to tandem structures, the shift from single cation-based perovskites to mix cation perovskties, and the emergence of low temperature fabrication techniques has all without a doubt proved beneficial in enhancing the PCE/stability of PSCs, yet many challenges must be overcome before mass production. Among the latest trends adopted for performance improvement of PSCs such as solvent engineering, interface modifications, use of solar concentrators, incorporating scattering effects, spectral modification and use of novel materials, the use of 2D MXenes emerged as the most attractive and effective technique for real world applications. Introduction of 2D Ti_3_C_2_T*_x_* MXene material into the building blocks of PSCs are summarized in this short review. Recent advances in using MXene as ETL/HTL and electrode have been diligently combined and summarized. It can be safely concluded that the important parameters determining the successful incorporation of MXene materials in PSCs are their concentration, surface terminations, WF, the major constituent into which they are added and composition and thickness of the host constituent (ETL, perovskite layer, HTL).

In the majority of the cases, upon successful addition of 2D Ti_3_C_2_T*_x_* MXene, stability and performance of PSCs considerably improved, owing to their superior charge transport properties and WF modifications. Well-adjusted electrical properties improved charge transport/extraction dynamics and well aligned energy levels, uniform film growth, suppressed trap state density, reduced recombinations, optimized interface properties and good film quality can be expected after introducing 2D Ti_3_C_2_T*_x_* MXene materials into the perovskite layer or ETL of PSCs owing to the superior charge transport, perfect band alignment and WF adjustment of 2D Ti_3_C_2_T*_x_* MXene materials.

## Figures and Tables

**Table 1:** Summary of the works reporting the incorporation of MXene (Ti_3_C_2_T*_x_*) in PSCs.

Device Architecture	Application Mode	V_OC_ (V) Pristine/ Improved	J_SC_ (mA cm^−2^) Pristine/ Improved	FF (%) Pristine/ Improved	PCE (%) Pristine/ Improved	Enhancement in PCE (%)	Year	Ref.
ITO/SnO_2_/perovskite: Ti_3_C_2_T*_x_*/Spiro-MeOTAD/Au	Additive into the photoactive layer	1/ 1.03	20.67/ 22.26	75/ 76	15.54/ 17.41	12	2018	[57]
FTO/SnO_2_/perovskite: Ti_3_C_2_T*_x_*/Spiro-MeOTAD/Au	Additive into the photoactive layer	1.08/ 1.12	21.53/ 23.48	70.4/ 73.6	16.45/ 19.27	18	2021	[67]
FTO/c-TiO_2_ +MXene/ m-TiO_2_ + MXene/MXene/perovskite + MXene/ spiro-OMeTAD/Au	Additive into the photoactive layer and ETL	-/1.09	-/23.82	-/77.6	-/20.14	26.5	2019	[68]
c-TiO_2_/m-TiO_2_-TQD/TQD- perovskite/ Spiro-OMeTAD-Cu_1.8_S	Additive into the photoactive layer and ETL	-/1.13	-/23.64	-/77.5	18.36/ 21.72	18.18	2020	[71]
FTO/c-TiO_2_/ m-TiO_2_-2D MXene/ perovskite-0D Ti_3_C_2_ QDs/Spiro-OMeTAD/Au	Additive into the photoactive layer and ETL	0.927/-	19.6/-	66.0/-	12/ 17.1	42.5	2020	[72]
ITO/SnO_2_/(BA)_2_(MA)_4_Pb_5_I_16_-Ti_3_C_2_ MXene/Spiro-OMeTAD/Ag	Additive into the photoactive layer	1.09/ 1.11	18.84/ 20.87	66.7/ 67.8	13.69/ 15.71	14.7	2021	[73]
ITO/SnO_2_-Ti_3_C_2_ MXene/MAPbI_3_/Spiro-OMeTAD/ Ag	Additive in ETL	1.05/ 1.06	22.83/ 23.14	72/ 75	17.23/ 18.34	6.4	2019	[74]
FTO/SnO_2_-MXene/ (FAPbI_3_)_0.97_(MAPbBr_3_)_0.03_/spiro-OMeTAD	Additive in ETL	1.07/ 1.1	24.07/ 24.52	73.6/ 77.9	16.83/ 19.14	13.7	2020	[75]
ITO/SnO_2_-MQDs/perovskite/ Spiro/MoO_3_/Au	Additive in ETL	1.14/ 1.17	24.26/ 24.96	75.8/ 79.8	20.96/ 23.34	11.35	2021	[76]
FTO/Ti_3_C_2_T*_x_*@TiO_2_ (0.2 wt%)/Cs_2_AgBiBr_6_/Spiro/MoO_3_/Ag	Additive in ETL	0.93/ 0.96	3.29/ 4.14	65/ 70	2.0/ 2.81	40.5	2021	[77]
NiO/MAPbI_3_/PCBM/BCP/Ag	Additive into the photoactive layer and ETL	1.09/ 1.09	21.41/ 22.88	77/ 77	17.97/ 19.20	8.3	2021	[78]
FTO/TiO_2_/CsPbBr_3_/ Ti_3_C_2_-MXene/C	As HTL	1.423/ 1.444	7.96/ 8.54	72.48/ 73.08	8.21/ 9.01	9.7	2019	[82]
ITO/Ti_3_C_2_T*_x_*/CH_3_NH_3_PbI_3_/ Spiro-OMeTAD/Ag	As ETL	-/1.08	-/22.63	-/70	-/17.17	-	2019	[83]
ITO/HO-Ti_3_C_2_T*_x_*@Ti_3_C_2_T*_x_*/CH_3_NH_3_PbI_3_/ Spiro-OMeTAD/Ag	As ETL	-/1.07	-/23.11	-/74	-/18.29	23	2021	[85]
FTO/MXene-SnO_2_/Perovskite/Spiro-OMeTAD/Au	As separate layer between SnO_2_ and FTO	-/1.11	-/24.34	-	19/ 20.65	8.6	2020	[84]
ITO/SnO_2_/ MXene:m-SWCNTs(2:1)/Perovskite/Spiro-OMeTAD/Au	As separate layer between perovskite and SnO_2_	1.043/ 1.073	24.71/ 25.09	73/ 80	18.84/ 21.42	13.7	2021	[86]
FTO/TiO_2_/CH_3_NH_3_PbI_3_/ MXene	As electrode	0.84/ 0.95	21.39/ 22.96	60/ 63	10.87/ 13.83	27	2019	[94]
FTO/c-TiO_2_/CsPbBr_3_/C+ CNTs+MXene	As electrode	1.250/ 1.357	5.81/ 7.16	65.68/ 72.97	4.77/ 7.09	48.6	2020	[95]

## References

[1] Green M.A., Dunlop E.D., Hohl-Ebinger J., Yoshita M., Kopidakis N., Hao X. (2021). Solar cell efficiency tables (Version 58). Prog. Photovolt. Res. Appl..

[2] Punathil L., Mohanasundaram K., Tamilselavan K.S., Sathyamurthy R., Chamkha A.J. (2021). Recovery of Pure Silicon and Other Materials from Disposed Solar Cells. Int. J. Photoenergy.

[3] Chowdhury M.S., Rahman K.S., Chowdhury T., Nuthammachot N., Techato K., Akhtaruzzaman M., Tiong S.K., Sopian K., Amin N. (2020). An overview of solar photovoltaic panels’ end-of-life material recycling. Energy Strategy Rev..

[4] Shah S.A.A., Sayyad M.H., Sun J., Guo Z. (2021). Hysteresis Analysis of Hole-Transport-Material-Free Monolithic Perovskite Solar Cells with Carbon Counter Electrode by Current Density–Voltage and Impedance Spectra Measurements. Nanomaterials.

[5] Shah S.A.A., Sayyad M.H., Khan K., Guo K., Shen F., Sun J., Tareen A.K., Gong Y., Guo Z. (2020). Progress towards High-Efficiency and Stable Tin-Based Perovskite Solar Cells. Energies.

[6] Li Y., Ji L., Liu R., Zhang C., Mak C.H., Zou X., Shen H.-H., Leu S.-Y., Hsu H.-Y. (2018). A review on morphology engineering for highly efficient and stable hybrid perovskite solar cells. J. Mater. Chem. A.

[7] Wu Y., Wang D., Liu J., Cai H. (2021). Review of Interface Passivation of Perovskite Layer. Nanomaterials.

[8] Li B., Zhang Y., Fu L., Zhang L., Liu Z., Yin L. (2019). Two-dimensional black phosphorous induced exciton dissociation efficiency enhancement for high-performance all-inorganic CsPbI3 perovskite photovoltaics. J. Mater. Chem. A.

[9] Wang H., Chan C.C.S., Chu M., Xie J., Zhao S., Guo X., Miao Q., Wong K.S., Yan K., Xu J. (2020). Interlayer Cross-Linked 2D Perovskite Solar Cell with Uniform Phase Distribution and Increased Exciton Coupling. Solar RRL.

[10] Wu T., Liu X., Luo X., Lin X., Cui D., Wang Y., Segawa H., Zhang Y., Han L. (2021). Lead-free tin perovskite solar cells. Joule.

[11] Juang S.S.-Y., Lin P.-Y., Lin Y.-C., Chen Y.-S., Shen P.-S., Guo Y.-L., Wu Y.-C., Chen P. (2019). Energy Harvesting Under Dim-Light Condition With Dye-Sensitized and Perovskite Solar Cells. Front. Chem..

[12] Kim H.-S., Lee C.-R., Im J.-H., Lee K.-B., Moehl T., Marchioro A., Moon S.-J., Humphry-Baker R., Yum J.-H., Moser J.E. (2012). Lead Iodide Perovskite Sensitized All-Solid-State Submicron Thin Film Mesoscopic Solar Cell with Efficiency Exceeding 9%. Sci. Rep..

[13] Yoo J.J., Seo G., Chua M.R., Park T.G., Lu Y., Rotermund F., Kim Y.-K., Moon C.S., Jeon N.J., Correa-Baena J.-P. (2021). Efficient perovskite solar cells via improved carrier management. Nature.

[14] Zhang F., Zhu K. (2020). Additive Engineering for Efficient and Stable Perovskite Solar Cells. Adv. Energy Mater..

[15] Park N.-G. (2020). Research Direction toward Scalable, Stable, and High Efficiency Perovskite Solar Cells. Adv. Energy Mater..

[16] Pazos-Outón L.M., Xiao T.P., Yablonovitch E. (2018). Fundamental Efficiency Limit of Lead Iodide Perovskite Solar Cells. J Phys. Chem. Lett..

[17] Brenes R., Laitz M., Jean J., deQuilettes D.W., Bulović V. (2019). Benefit from Photon Recycling at the Maximum-Power Point of State-of-the-Art Perovskite Solar Cells. Phys. Rev. Applied.

[18] Wang D., Wright M., Elumalai N.K., Uddin A. (2016). Stability of perovskite solar cells. Sol. Energy Mater. Sol. Cells.

[19] de la Mora M.B., Amelines-Sarria O., Monroy B.M., Hernández-Pérez C.D., Lugo J.E. (2017). Materials for downconversion in solar cells: Perspectives and challenges. Sol. Energy Mater. Sol. Cells.

[20] Kakavelakis G., Petridis K., Kymakis E. (2017). Recent advances in plasmonic metal and rare-earth-element upconversion nanoparticle doped perovskite solar cells. J. Mater. Chem. A.

[21] Li J., Aierken A., Liu Y., Zhuang Y., Yang X., Mo J.H., Fan R.K., Chen Q.Y., Zhang S.Y., Huang Y.M. (2021). A Brief Review of High Efficiency III-V Solar Cells for Space Application. Front. Phys..

[22] Emetere M.E., Emetere J.M., Ometan O.O. (2019). A short review on solar concentrator for energy generation in tropical coastal belt. J. Phys. Conf. Ser..

[23] Li M., Begum R., Fu J., Xu Q., Koh T.M., Veldhuis S.A., Grätzel M., Mathews N., Mhaisalkar S., Sum T.C. (2018). Low threshold and efficient multiple exciton generation in halide perovskite nanocrystals. Nat. Commun..

[24] Day J., Senthilarasu S., Mallick T.K. (2019). Improving spectral modification for applications in solar cells: A review. Renewable Energy.

[25] Luceño-Sánchez J.A., Díez-Pascual A.M., Peña Capilla R. (2019). Materials for Photovoltaics: State of Art and Recent Developments. Int. J. Mol. Sci..

[26] Sai Gautam G., Senftle T.P., Alidoust N., Carter E.A. (2018). Novel Solar Cell Materials: Insights from First-Principles. J. Phys. Chem. C.

[27] Li M., Li H., Fu J., Liang T., Ma W. (2020). Recent Progress on the Stability of Perovskite Solar Cells in a Humid Environment. J. Phys. Chem. C.

[28] Dagar J., Fenske M., Al-Ashouri A., Schultz C., Li B., Köbler H., Munir R., Parmasivam G., Li J., Levine I. (2021). Compositional and Interfacial Engineering Yield High-Performance and Stable p-i-n Perovskite Solar Cells and Mini-Modules. ACS Appl. Mater. Interfaces.

[29] Yin L., Li Y., Yao X., Wang Y., Jia L., Liu Q., Li J., Li Y., He D. (2021). MXenes for Solar Cells. Nano-Micro Lett..

[30] Sui J., Chen X., Li Y., Peng W., Zhang F., Fan X. (2021). MXene derivatives: Synthesis and applications in energy convention and storage. RSC Adv..

[31] Garg R., Agarwal A., Agarwal M. (2020). A review on MXene for energy storage application: Effect of interlayer distance. Mater. Res. Exp..

[32] Das P., Wu Z.-S. (2020). MXene for energy storage: Present status and future perspectives. J. Phys. Energy.

[33] Anasori B., Lukatskaya M.R., Gogotsi Y. (2017). 2D metal carbides and nitrides (MXenes) for energy storage. Nat. Rev. Mater..

[34] Li X., Huang Z., Zhi C. (2019). Environmental Stability of MXenes as Energy Storage Materials. Front. Mater..

[35] Morales-García Á., Calle-Vallejo F., Illas F. (2020). MXenes: New Horizons in Catalysis. ACS Catalysis.

[36] Gao G., O’Mullane A.P., Du A. (2017). 2D MXenes: A New Family of Promising Catalysts for the Hydrogen Evolution Reaction. ACS Catal..

[37] Li Z., Yu L., Milligan C., Ma T., Zhou L., Cui Y., Qi Z., Libretto N., Xu B., Luo J. (2018). Two-dimensional transition metal carbides as supports for tuning the chemistry of catalytic nanoparticles. Nat. Commun..

[38] Gouveia J.D., Morales-García Á., Viñes F., Illas F., Gomes J.R.B. (2020). MXenes as promising catalysts for water dissociation. Appl. Catal. B.

[39] Sun J., Kong W., Jin Z., Han Y., Ma L., Ding X., Niu Y., Xu Y. (2020). Recent advances of MXene as promising catalysts for electrochemical nitrogen reduction reaction. Chin. Chem. Lett..

[40] Zhou S., Yang X., Pei W., Jiang Z., Zhao J. (2020). MXene and MBene as efficient catalysts for energy conversion: Roles of surface, edge and interface. J. Phys. Energy.

[41] Zamhuri A., Lim G.P., Ma N.L., Tee K.S., Soon C.F. (2021). MXene in the lens of biomedical engineering: Synthesis, applications and future outlook. Biomed. Eng. Online.

[42] George S.M., Kandasubramanian B. (2020). Advancements in MXene-Polymer composites for various biomedical applications. Ceram. Int..

[43] Lin H., Chen Y., Shi J. (2018). Insights into 2D MXenes for Versatile Biomedical Applications: Current Advances and Challenges Ahead. Adv. Sci..

[44] Soleymaniha M., Shahbazi M.-A., Rafieerad A.R., Maleki A., Amiri A. (2019). Promoting Role of MXene Nanosheets in Biomedical Sciences: Therapeutic and Biosensing Innovations. Adv. Healthc. Mater..

[45] Vitale F., Driscoll N., Murphy B., Anasori B., Gogotsi Y. (2019). Biomedical Applications of MXenes. 2D Metal Carbides and Nitrides (MXenes): Structure, Properties and Applications.

[46] Jimmy J., Kandasubramanian B. (2020). Mxene functionalized polymer composites: Synthesis and applications. Eur. Polym. J..

[47] Li K., Lei Y., Liao J., Zhang Y. (2021). Facile synthesis of MXene-supported copper oxide nanocomposites for catalyzing the decomposition of ammonium perchlorate. Inorg. Chem. Front..

[48] Bora P.J., Anil A.G., Ramamurthy P.C., Tan D.Q. (2020). MXene interlayered crosslinked conducting polymer film for highly specific absorption and electromagnetic interference shielding. Mater. Adv..

[49] Guo J., Legum B., Anasori B., Wang K., Lelyukh P., Gogotsi Y., Randall C.A. (2018). Cold Sintered Ceramic Nanocomposites of 2D MXene and Zinc Oxide. Adv. Mater..

[50] Liang L., Li Q., Yan X., Feng Y., Wang Y., Zhang H.-B., Zhou X., Liu C., Shen C., Xie X. (2021). Multifunctional Magnetic Ti3C2Tx MXene/Graphene Aerogel with Superior Electromagnetic Wave Absorption Performance. ACS Nano.

[51] Liu J., Zhang H.B., Sun R., Liu Y., Liu Z., Zhou A., Yu Z.Z. (2017). Hydrophobic, Flexible, and Lightweight MXene Foams for High-Performance Electromagnetic-Interference Shielding. Adv. Mater..

[52] Wan S., Li X., Wang Y., Chen Y., Xie X., Yang R., Tomsia A.P., Jiang L., Cheng Q. (2020). Strong sequentially bridged MXene sheets. Proc. Natl. Acad. Sci. USA.

[53] Lyu B., Kim M., Jing H., Kang J., Qian C., Lee S., Cho J.H. (2019). Large-Area MXene Electrode Array for Flexible Electronics. ACS Nano.

[54] Kim H., Alshareef H.N. (2020). MXetronics: MXene-Enabled Electronic and Photonic Devices. ACS Mater. Lett..

[55] Li N., Peng J., Ong W.J., Ma T., Zhang P., Jiang J., Yuan X., Zhang C.J. (2021). MXenes: An Emerging Platform for Wearable Electronics and Looking Beyond. Matter.

[56] Wang Y., Xu Y., Hu M., Ling H., Zhu X. (2020). MXenes: Focus on optical and electronic properties and corresponding applications. Nanophotonics.

[57] Guo Z., Gao L., Xu Z., Teo S., Zhang C., Kamata Y., Hayase S., Ma T. (2018). High Electrical Conductivity 2D MXene Serves as Additive of Perovskite for Efficient Solar Cells. Small.

[58] Bati A.S.R., Batmunkh M., Shapter J.G. (2020). Emerging 2D Layered Materials for Perovskite Solar Cells. Adv. Energy Mater..

[59] Li S., Cao Y.-L., Li W.-H., Bo Z.-S. (2021). A brief review of hole transporting materials commonly used in perovskite solar cells. Rare Met..

[60] Valadi K., Gharibi S., Taheri-Ledari R., Akin S., Maleki A., Shalan A.E. (2021). Metal oxide electron transport materials for perovskite solar cells: A review. Environ. Chem. Lett..

[61] Park N.-G., Zhu K. (2020). Scalable fabrication and coating methods for perovskite solar cells and solar modules. Nat. Rev. Mater..

[62] Hadadian M., Correa-Baena J.P., Goharshadi E.K., Ummadisingu A., Seo J.Y., Luo J., Gholipour S., Zakeeruddin S.M., Saliba M., Abate A. (2016). Enhancing Efficiency of Perovskite Solar Cells via N-doped Graphene: Crystal Modification and Surface Passivation. Adv. Mater..

[63] Jiang L.-L., Wang Z.-K., Li M., Zhang C.-C., Ye Q.-Q., Hu K.-H., Lu D.-Z., Fang P.-F., Liao L.-S. (2018). Passivated Perovskite Crystallization via g-C3N4 for High-Performance Solar Cells. Adv. Funct. Mater..

[64] Ma C., Shi Y., Hu W., Chiu M.H., Liu Z., Bera A., Li F., Wang H., Li L.J., Wu T. (2016). Heterostructured WS2/CH3 NH3 PbI3 Photoconductors with Suppressed Dark Current and Enhanced Photodetectivity. Adv. Mater..

[65] Capasso A., Matteocci F., Najafi L., Prato M., Buha J., Cinà L., Pellegrini V., Carlo A.D., Bonaccorso F. (2016). Few-Layer MoS2 Flakes as Active Buffer Layer for Stable Perovskite Solar Cells. Adv. Energy Mater..

[66] Chen W., Li K., Wang Y., Feng X., Liao Z., Su Q., Lin X., He Z. (2017). Black Phosphorus Quantum Dots for Hole Extraction of Typical Planar Hybrid Perovskite Solar Cells. J. Phys. Chem. Lett..

[67] Zhao Y., Zhang X., Han X., Hou C., Wang H., Qi J., Li Y., Zhang Q. (2021). Tuning the reactivity of PbI2 film via monolayer Ti3C2Tx MXene for two-step-processed CH3NH3PbI3 solar cells. Chem. Eng. J..

[68] Agresti A., Pazniak A., Pescetelli S., Di Vito A., Rossi D., Pecchia A., Auf der Maur M., Liedl A., Larciprete R., Kuznetsov D.V. (2019). Titanium-carbide MXenes for work function and interface engineering in perovskite solar cells. Nat. Mater..

[69] Di Vito A., Pecchia A., Auf der Maur M., Di Carlo A. (2020). Nonlinear Work Function Tuning of Lead-Halide Perovskites by MXenes with Mixed Terminations. Adv. Funct. Mater..

[70] Zhang Z., Li Y., Liang C., Yu G., Zhao J., Luo S., Huang Y., Su C., Xing G. (2020). In Situ Growth of MAPbBr3 Nanocrystals on Few-Layer MXene Nanosheets with Efficient Energy Transfer. Small.

[71] Chen X., Xu W., Ding N., Ji Y., Pan G., Zhu J., Zhou D., Wu Y., Chen C., Song H. (2020). Dual Interfacial Modification Engineering with 2D MXene Quantum Dots and Copper Sulphide Nanocrystals Enabled High-Performance Perovskite Solar Cells. Adv. Funct. Mater..

[72] Ge J., Li W., He X., Chen H., Fang W., Du X., Li Y., Zhao L. (2020). Charge behavior modulation by titanium-carbide quantum dots and nanosheets for efficient perovskite solar cells. Mater. Today Energy.

[73] Jin X., Yang L., Wang X.-F. (2021). Efficient Two-Dimensional Perovskite Solar Cells Realized by Incorporation of Ti3C2Tx MXene as Nano-Dopants. Nano-micro Lett..

[74] Yang L., Dall’Agnese Y., Hantanasirisakul K., Shuck C.E., Maleski K., Alhabeb M., Chen G., Gao Y., Sanehira Y., Jena A.K. (2019). SnO2–Ti3C2 MXene electron transport layers for perovskite solar cells. J. Mater. Chem. A.

[75] Huang L., Zhou X., Xue R., Xu P., Wang S., Xu C., Zeng W., Xiong Y., Sang H., Liang D. (2020). Low-Temperature Growing Anatase TiO2/SnO2 Multi-dimensional Heterojunctions at MXene Conductive Network for High-Efficient Perovskite Solar Cells. Nano-Micro Lett..

[76] Yang Y., Lu H., Feng S., Yang L., Dong H., Wang J., Tian C., Li L., Lu H., Jeong J. (2021). Modulation of perovskite crystallization processes towards highly efficient and stable perovskite solar cells with MXene quantum dot-modified SnO2. Energy Environ. Sci..

[77] Li Z., Wang P., Ma C., Igbari F., Kang Y., Wang K.-L., Song W., Dong C., Li Y., Yao J. (2021). Single-Layered MXene Nanosheets Doping TiO2 for Efficient and Stable Double Perovskite Solar Cells. J. Am. Chem. Soc..

[78] Saranin D., Pescetelli S., Pazniak A., Rossi D., Liedl A., Yakusheva A., Luchnikov L., Podgorny D., Gostischev P., Didenko S. (2021). Transition metal carbides (MXenes) for efficient NiO-based inverted perovskite solar cells. Nano Energy.

[79] Hou C., Yu H. (2020). Modifying the nanostructures of PEDOT:PSS/Ti3C2TX composite hole transport layers for highly efficient polymer solar cells. J. Mater. Chem. C.

[80] Pan H., Zhao X., Gong X., Li H., Ladi N.H., Zhang X.L., Huang W., Ahmad S., Ding L., Shen Y. (2020). Advances in design engineering and merits of electron transporting layers in perovskite solar cells. Mater. Horizons.

[81] Shao H., Ladi N.H., Pan H., Zhang X.L., Shen Y., Wang M. (2021). 2D Materials as Electron Transport Layer for Low-Temperature Solution-Processed Perovskite Solar Cells. Solar RRL.

[82] Chen T., Tong G., Xu E., Li H., Li P., Zhu Z., Tang J., Qi Y., Jiang Y. (2019). Accelerating hole extraction by inserting 2D Ti3C2-MXene interlayer to all inorganic perovskite solar cells with long-term stabilityJ. Mater. Chem. A.

[83] Yang L., Dall’Agnese C., Dall’Agnese Y., Chen G., Gao Y., Sanehira Y., Jena A.K., Wang X.-F., Gogotsi Y., Miyasaka T. (2019). Surface-Modified Metallic Ti3C2Tx MXene as Electron Transport Layer for Planar Heterojunction Perovskite Solar Cells. Adv. Funct. Mater..

[84] Wang Y., Xiang P., Ren A., Lai H., Zhang Z., Xuan Z., Wan Z., Zhang J., Hao X., Wu L. (2020). MXene-Modulated Electrode/SnO2 Interface Boosting Charge Transport in Perovskite Solar Cells. ACS Appl. Mater. Interfaces.

[85] Yang L., Kan D., Dall’Agnese C., Dall’Agnese Y., Wang B., Jena A.K., Wei Y., Chen G., Wang X.-F., Gogotsi Y. (2021). Performance improvement of MXene-based perovskite solar cells upon property transition from metallic to semiconductive by oxidation of Ti3C2Tx in air. J. Mater. Chem. A.

[86] Bati A.S.R., Hao M., Macdonald T.J., Batmunkh M., Yamauchi Y., Wang L., Shapter J.G. (2021). 1D-2D Synergistic MXene-Nanotubes Hybrids for Efficient Perovskite Solar Cells. Small.

[87] Wei J., Xu R.-P., Li Y.-Q., Li C., Chen J.-D., Zhao X.-D., Xie Z.-Z., Lee C.-S., Zhang W.-J., Tang J.-X. (2017). Enhanced Light Harvesting in Perovskite Solar Cells by a Bioinspired Nanostructured Back Electrode. Adv. Energy Mater..

[88] Tran V.-D., Pammi S.V.N., Park B.-J., Han Y., Jeon C., Yoon S.-G. (2019). Transfer-free graphene electrodes for super-flexible and semi-transparent perovskite solar cells fabricated under ambient air. Nano Energy.

[89] Bogachuk D., Zouhair S., Wojciechowski K., Yang B., Babu V., Wagner L., Xu B., Lim J., Mastroianni S., Pettersson H. (2020). Low-temperature carbon-based electrodes in perovskite solar cells. Energy Environ. Sci..

[90] Liu Z., He H., Atesin T.A., Bashir S., Liu J.L. (2019). Counter Electrode Materials for Organic-Inorganic Perovskite Solar Cells. Nanostructured Materials for Next-Generation Energy Storage and Conversion: Photovoltaic and Solar Energy.

[91] Zhang J., Kong N., Uzun S., Levitt A., Seyedin S., Lynch P.A., Qin S., Han M., Yang W., Liu J. (2020). Scalable Manufacturing of Free-Standing, Strong Ti3C2Tx MXene Films with Outstanding Conductivity. Adv. Mater..

[92] Hantanasirisakul K., Gogotsi Y. (2018). Electronic and Optical Properties of 2D Transition Metal Carbides and Nitrides (MXenes). Adv. Mater..

[93] Ali G., Iqbal M.Z., Jan Iftikhar F., Arshid N., Khalid M., Grace A.N. (2021). Chapter ten—MXene. Advances in Supercapacitor and Supercapattery.

[94] Cao J., Meng F., Gao L., Yang S., Yan Y., Wang N., Liu A., Li Y., Ma T. (2019). Alternative electrodes for HTMs and noble-metal-free perovskite solar cells: 2D MXenes electrodes. RSC Adv..

[95] Mi L., Zhang Y., Chen T., Xu E., Jiang Y. (2020). Carbon electrode engineering for high efficiency all-inorganic perovskite solar cells. RSC Adv..

